# The Ubiquitin–Proteasome System in Flowering Plant Reproduction: Mechanisms, Functional Diversity, and Regulatory Networks

**DOI:** 10.3390/plants15101433

**Published:** 2026-05-08

**Authors:** Xiaohu Jiang, Han Su, Mengnan Chai, Fan Yang, Hanyang Cai, Yuan Qin, Maokai Yan

**Affiliations:** College of Life Sciences, Haixia Institute of Science and Technology, School of Future Technology, Fujian Agriculture and Forestry University, Fuzhou 350002, China; jiangxiaohu6754@163.com (X.J.); suhan@fafu.edu.cn (H.S.); chaimengnan1@163.com (M.C.); atlas_yf@163.com (F.Y.); caihanyang8816@fafu.edu.cn (H.C.)

**Keywords:** female germline, male germline, E3 ubiquitin ligase, ubiquitin–proteasome system, embryogenesis

## Abstract

The ubiquitin–proteasome system (UPS) is a highly conserved protein degradation pathway in eukaryotic cells. Through precisely controlled proteolysis of key regulatory proteins, the UPS plays a particularly critical role in plant sexual reproduction, where precise spatiotemporal regulation is essential. The UPS governs multiple aspects of plant sexual reproduction, including male and female gametophyte development, pollen–pistil interactions, double fertilization, and post-fertilization embryogenesis and endosperm development. Among UPS components, E3 ubiquitin ligases play a central role by mediating the spatiotemporal degradation of key proteins, while E2 conjugating enzymes and deubiquitinating enzymes also make essential contributions. Through cross-species and cross-stage comparisons, we find that the UPS exhibits conserved regulatory logic—including cell-cycle gating, spatial control of protein accumulation, and signal integration—while also having evolved lineage-specific functional diversification. In this review, we systematically synthesize UPS functions across the reproductive cycle and highlight persistent knowledge gaps, aiming to provide an integrated framework and a reference for future studies investigating the regulatory roles of the UPS in plant sexual reproduction.

## 1. Introduction

Sexual reproduction is the predominant mode of reproduction in land plants and constitutes a critical phase in the life cycle of seed plants. Its successful completion depends not only on the proper morphogenesis of male and female reproductive organs but also on the precise spatiotemporal coordination of pollination, fertilization, and subsequent embryogenesis [1]. Within seed plants, angiosperms (flowering plants) represent the vast majority of extant species, and it is estimated that approximately 90% of them rely on sexual reproduction [2]. This high prevalence underscores the essential role of functional female and male gametophyte development in ensuring reproductive success in angiosperms [2].

Male gametophyte development in angiosperms is initiated by the specification of archesporial cells within the anther. These cells undergo a periclinal division, giving rise to an outer primary parietal cell and an inner primary sporogenous cell. The sporogenous cells ultimately differentiate into microspore mother cells (MMCs), also referred to as pollen mother cells (PMCs) [3]. In transverse section, the anther locule containing the PMCs is surrounded by several concentric cell layers: the tapetum, middle layer, endothecium, and epidermis, arranged from the innermost to the outermost [4,5]. During development, the middle layer is typically ephemeral and degrades, while the tapetum undergoes programmed cell death at later stages to nourish the developing microspores. The PMCs undergo meiosis to yield four haploid microspores, initially enclosed as a tetrad. Subsequently, each microspore undergoes an asymmetric mitotic division (pollen mitosis I, PMI), forming a bicellular pollen grain composed of a vegetative cell and a generative cell [6,7]. The generative cell then undergoes a second mitotic division (pollen mitosis II, PMII) to produce two sperm cells. Mature pollen grains are classified into two types—bicellular and tricellular—based on the timing of this second mitotic division [8]. In bicellular pollen, division of the generative cell is delayed until after pollination and occurs during pollen tube growth; consequently, the mature pollen grain at dispersal contains only a vegetative cell and a generative cell. In contrast, in tricellular pollen, the generative cell division is completed prior to anthesis, and the mature pollen grain consists of a vegetative cell and two sperm cells [9].

The female gametophyte of flowering plants originates from somatic cells within the ovule [10]. Its development proceeds through two major phases: megasporogenesis and female gametogenesis [11]. Megasporogenesis is initiated in the nucellus, where a single subepidermal cell beneath the nucellar apex differentiates into an archesporial cell (AC). Following cellular expansion and differentiation, the AC gives rise to the megaspore mother cell (MMC). The MMC then undergoes meiosis, producing four haploid megaspores. As development proceeds, the three megaspores at the micropylar end degenerate, leaving only the chalazal megaspore to survive as the functional megaspore. Subsequently, this functional megaspore enters female gametogenesis, undergoing three rounds of mitotic divisions to form an eight-nucleate coenocyte. Following cellularization, it ultimately develops into the mature female gametophyte (the embryo sac), which comprises seven cells and eight nuclei [12,13,14].

The development of male and female gametophytes, a critical phase in plant sexual reproduction, is critically dependent on a tightly regulated balance between protein synthesis and degradation. In angiosperms, the progression from sporogenesis to mature gametophyte formation is orchestrated by the spatiotemporally specific expression of various regulatory proteins—including transcription factors, cell cycle regulators, and signaling components—that precisely drive developmental transitions. The stringent spatiotemporal precision required for gametophyte development renders protein homeostasis particularly crucial: aberrant accumulation of key regulators—resulting from either dysregulated synthesis or impaired degradation—can disrupt cell cycle progression, interfere with polarity establishment, or induce abnormal programmed cell death. Such perturbations ultimately lead to structural aberrations in the gametophyte, severely compromising double fertilization efficiency and seed fertility. To counter these challenges, plants have evolved multilayered protein quality control (PQC) systems, such as the ubiquitin–proteasome pathway to surveil and maintain the proteome.

The UPS serves as a core proteolytic pathway [15], and is widely involved in regulating plant growth, development, and adaptation to environmental stimuli [16,17]. This system operates through a coordinated cascade involving ubiquitin-activating (E1), -conjugating (E2), and -ligating (E3) enzymes, which ultimately label target substrates with ubiquitin chains for degradation by the 26S proteasome [18]. Additional regulators, such as deubiquitinating enzymes and specific shuttle factors, further refine the process. Within this system, E3 ubiquitin ligases function as central regulatory elements. Through their diverse structural domains—such as RING, HECT, or F-box motifs—they confer substrate specificity, thereby playing a decisive role in the ubiquitination cascade [19]. To facilitate a comprehensive and comparative understanding of the diverse UPS components implicated in plant reproduction, Table 1 summarizes the key factors discussed in this review. The table categorizes each component by UPS type, reproductive process, specific developmental stage, substrate identity and species of origin. This systematic organization not only highlights the breadth of UPS involvement across male and female gametogenesis, pollen–pistil interactions, and reproductive barriers, but also reveals notable gaps in our current knowledge, such as the preponderance of genetically characterized but biochemically unvalidated E3 ligases in female reproductive tissues. This review not only systematically summarizes specific examples of UPS functions in male gametophyte, female gametophyte, gametophyte interactions and post-fertilization embryogenesis and endosperm development, but also aims to extract the underlying common regulatory logic (e.g., cyclin-dependent degradation, quality control pathways), lineage-specific adaptations (e.g., differences between monocots and dicots), and broader regulatory themes (e.g., hormone signaling integration, environmental responses, and evolutionary isolation).

The literature discussed in this review was identified through targeted searches in PubMed, Google Scholar, and Web of Science Core Collection, using core keywords including “ubiquitin-proteasome,” “E3 ubiquitin ligase,” “deubiquitinating enzyme,” “plant reproduction,” “gametophyte,” “pollen,” “self-incompatibility,” and “embryogenesis,” with an emphasis on peer-reviewed research articles and high-impact reviews published between 2000 and 2026. Studies were prioritized that provide genetic, biochemical, or cell biological evidence for specific UPS components with demonstrated functions in flowering plant reproductive development. Additional foundational studies were identified through citation tracing.

## 2. The Ubiquitin–Proteasome System: Core Components and Mechanisms

In eukaryotic cells, protein degradation is primarily mediated by two mechanisms: the UPS and autophagy [50]. The UPS is responsible for the selective, ubiquitin-tagged degradation of short-lived regulatory proteins and misfolded polypeptides. Autophagy, by contrast, mediates the bulk degradation of long-lived proteins, protein aggregates, and entire organelles through vacuolar or lysosomal delivery [51]. Together, these two pathways maintain cellular protein homeostasis—the UPS provides precise spatiotemporal control over specific regulatory proteins [52], while autophagy handles large-scale turnover and quality control of cytoplasmic components [53]. Both pathways are essential for plant development and stress responses, and their relative contributions vary across tissues and developmental stages.

Among these, the UPS constitutes the primary pathway for the selective and regulated degradation of proteins, governing the turnover of the majority of short-lived and misfolded proteins under physiological conditions [54]. This system is essential for maintaining cellular protein homeostasis by orchestrating both the precise elimination of specific regulatory proteins and the clearance of damaged or aberrant polypeptides [55]. The central player in this pathway is ubiquitin, a small (~8.5 kDa), highly conserved globular protein of 76 amino acids. Its amino acid sequence exhibits over 95% identity among eukaryotes. Structurally, ubiquitin adopts a characteristic compact “β-grasp” fold, composed of a five-stranded β-sheet that cradles a single α-helix. Full-length ubiquitin possesses seven lysine residues—K6, K11, K27, K29, K33, K48, and K63—each capable of serving as a linkage point for chain elongation, as well as the N-terminal methionine (M1) [56,57]. This multiplicity of linkage options generates polyubiquitin chains with distinct topologies that encode different functional messages [58]. K48-linked chains represent the canonical signal for proteasomal degradation, directing modified proteins to the 26S proteasome for turnover—a function extensively illustrated throughout this review [58]. In contrast, K63-linked chains typically serve non-proteolytic roles, including membrane protein endocytosis, DNA damage repair, and activation of signaling cascades [57]. Beyond these canonical linkages, atypical chains such as K11-linked chains also participate in cell-cycle regulation and developmental processes [59]. As discussed in subsequent sections, these distinct chain types are differentially deployed across reproductive stages and tissues, underscoring that the UPS governs plant reproduction not only through the presence or absence of ubiquitination, but also through the specific topology of the ubiquitin chains assembled.

**Figure 1:**
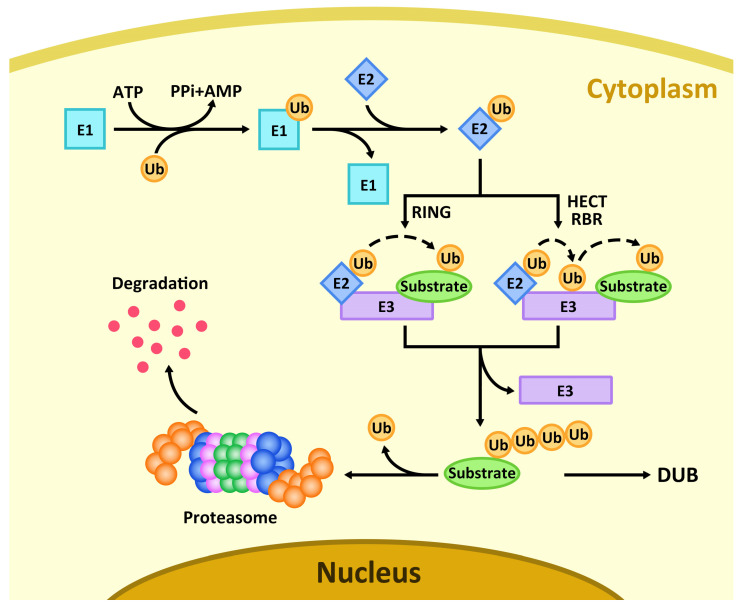
The ubiquitin cascade and chain formation. Ubiquitin (Ub) is activated by the E1 enzyme in an ATP-dependent manner to form an E1~Ub thioester intermediate. Ub is then transferred to the active-site cysteine of an E2-conjugating enzyme, yielding an E2~Ub thioester intermediate. Subsequently, Ub is conjugated to target proteins via two distinct pathways mediated by E3 ligases, which fall into three classes: RING, HECT, and RBR. RING E3s act as molecular scaffolds that simultaneously bind the substrate and the E2~Ub conjugate, facilitating the direct transfer of Ub from the E2 active site to a lysine residue on the substrate. In contrast, HECT and RBR E3s contain an active-site cysteine that forms a thioester-linked Ub-E3 intermediate before catalyzing the covalent attachment of Ub to the lysine residues of the specifically recruited substrate. Finally, most ubiquitinated proteins are recognized and degraded by the 26S proteasome, which releases free Ub for subsequent recycling.

The ubiquitin–proteasome pathway is initiated by the ATP-dependent activation of Ub by the E1 activating enzyme. This occurs in two steps: first, the C-terminal carboxyl group of ubiquitin is adenylated, and then it reacts with the catalytic cysteine of E1 to form a high-energy thioester bond. Subsequently, Ub is transferred to the catalytic cysteine of a ubiquitin-conjugating enzyme (E2) via a transthiolation reaction, forming a Ub~E2 thioester intermediate. Finally, a ubiquitin ligase (E3) facilitates the transfer of ubiquitin from the E2 to the target protein. This process is repeated to form a polyubiquitin chain, which serves as the canonical signal for recognition and degradation by the 26S proteasome [60,61]. Notably, the final transfer of ubiquitin to a substrate lysine occurs through one of two distinct mechanisms, which are determined by the E3 ligase class [62]. In the first pathway, employed by RING (Really Interesting New Gene) E3 ligases, the E3 acts as a scaffold that simultaneously binds both the substrate and a ubiquitin-charged E2 (E2~Ub). This positioning promotes the direct transfer of ubiquitin from the E2 active site to the ε-amino group of a lysine residue on the substrate, forming an isopeptide bond. In this case, no E3~Ub intermediate is formed. In the second pathway, utilized by HECT (Homologous to E6-associated Carboxy-Terminus) and RBR (RING-Between-RING) E3 ligases, ubiquitin is first transferred from the E2~Ub to an active-site cysteine on the E3 itself via a transthiolation reaction, generating a Ub~E3 thioester intermediate. The E3, already bound to its substrate via a separate domain, then catalyzes the transfer of ubiquitin from its own active site directly to the lysine residue on the substrate [21,63,64] (Figure 1).

Ubiquitination orchestrates diverse cellular signaling pathways by modulating protein activity and stability. Accurate substrate recognition is therefore essential for ensuring signal fidelity. E3 ligases play a pivotal role in ubiquitination by specifically recognizing target proteins [52]. The requirement for high substrate specificity has driven the remarkable proliferation of the E3 ligase gene family. For instance, in the model plant *Arabidopsis thaliana*, the UPS is encoded by only two E1 and 37 E2 enzymes, but more than 1500 E3 ligases—a striking amplification that accounts for over 95% of all UPS genes [64,65,66,67]. This phenomenon is conserved across eukaryotes; the genomes of Drosophila and humans contain approximately 100 and over 600 E3 ligase genes, respectively. This enormous diversity underscores the central role of E3 ligases in regulating proteostasis and reflects the intricate layer of specificity required to govern complex cellular signaling networks [68,69].

Based on their structural characteristics and mechanism of ubiquitin transfer, E3 ubiquitin ligases are broadly categorized into three major families: RING, HECT, and RBR types [70].

- RING-type E3 ubiquitin ligases act as scaffolds that facilitate direct transfer of ubiquitin from the E2 enzyme to the substrate. This family includes single-subunit RING ligases, U-box domain-containing ligases (a structural variant of RING), and the multi-subunit Cullin-RING Ligases (CRLs). CRLs assemble functional modules around a cullin scaffold and represent the largest class of E3 ligases, with primary categories including SCF (SKP1-Cullin1-F-box), CRL3 (utilizing BTB domain proteins), CRL4 (utilizing DDB1 adaptors), and the highly specialized APC/C.
- HECT-type E3 ubiquitin ligases form a catalytic thioester intermediate with ubiquitin before transferring it to the substrate.
- RBR-type E3 ubiquitin ligases combine features of both RING and HECT mechanisms. They contain RING domains to bind E2 enzymes but, like HECT ligases, they transfer ubiquitin via an obligate thioester intermediate formed on their own catalytic cysteine residue.

This hierarchical classification reflects the mechanistic diversity in substrate recognition and ubiquitin transfer, with the modular architecture of CRLs enabling broad substrate specificity through combinatorial assembly [71,72,73].

In addition to the ubiquitin conjugation machinery, deubiquitinating enzymes (DUBs) constitute an equally essential arm of the UPS. DUBs are a diverse collection of proteases that specifically cleave ubiquitin from conjugated substrates, performing three principal functions: (i) generating free ubiquitin monomers by processing the primary translation products of ubiquitin genes, which typically encode ubiquitin as fusions to itself or ribosomal proteins; (ii) recycling ubiquitin from polyubiquitinated proteins during or after proteasomal degradation to maintain the cellular pool of free ubiquitin; and (iii) reversing ubiquitination by removing ubiquitin moieties from specific targets, thereby antagonizing E3 ligase activity and modulating protein stability, activity, or localization [74,75]. The *Arabidopsis* genome encodes at least 27 ubiquitin-specific proteases (UBPs), which represent the largest DUB family in plants and are defined by signature Cys and His boxes that contain catalytically essential residues [76]. Additional DUB families include ubiquitin C-terminal hydrolases (UCHs), ovarian tumor domain proteases (OTUs), and the JAMM/MPN^+^ metalloproteases. The presence of dedicated deubiquitination machinery underscores that ubiquitination is a dynamic and reversible post-translational modification, and the coordinated action of E3 ligases and DUBs determines the net ubiquitination status of target proteins. As discussed in subsequent sections, specific DUBs have emerged as critical regulators of plant reproductive development, acting at distinct stages from gametogenesis to embryogenesis.

## 3. Role of the UPS in Male Gametophyte Development

**Figure 2:**
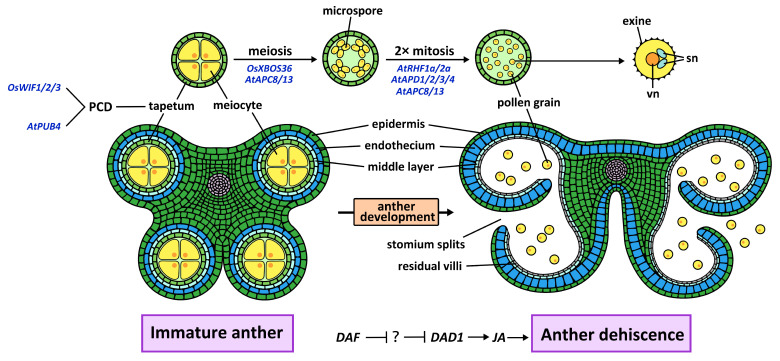
UPS component E3 ligase functions in male fertility. In angiosperms, male gametophyte development is precisely orchestrated by the ubiquitin–proteasome system (UPS) at distinct stages. The APC/C complex (APC8/APC13) functions during meiosis and the subsequent mitotic divisions; RHF1a/RHF2a act in post-meiotic mitotic progression at the microspore and bicellular stages; APD1-APD4 regulate the second pollen mitotic division (PMII). In rice, WIF1–WIF3 control the meiotic stage by mediating degradation of WA352 to prevent cytoplasmic male sterility, while XBOS36 targets MEL1 for degradation during meiosis; During anther development, AtPUB4 function in the tapetum to regulate pollen exine formation, and DAF acts at late stages to coordinate anther dehiscence via jasmonic acid biosynthesis. vn, vegetative nucleus; sn, sperm nucleus.

In angiosperms, the development of mature pollen grains from pollen mother cells proceeds through one meiotic and two mitotic divisions, a tightly regulated process (Figure 2). The Anaphase-Promoting Complex/Cyclosome (APC/C), the largest known E3 ubiquitin ligase, governs chromosome segregation during both mitosis and meiosis in animals [77,78,79]. This role is conserved in plants [80]. In *Arabidopsis*, the APC/C functions as an essential driver of cell-cycle progression in the male germline: loss of *APC8* or *APC13* disrupts meiotic chromosome segregation and arrests pollen mitosis, phenotypes linked to impaired degradation of mitotic cyclins such as CYCB1;1 [20,21,22]. However, direct biochemical evidence for this E3–substrate pair in *Arabidopsis* has not yet been reported. Neither in vitro ubiquitination assays nor detection of APC/C-dependent ubiquitination of CYCB1;1 in plant extracts has been documented. The abnormal accumulation of CYCB1;1 in *apc* mutants therefore provides strong genetic evidence consistent with impaired degradation, but whether CYCB1;1 is a direct ubiquitination target of the plant APC/C remains to be formally demonstrated. In rice, loss of *OsAPC6* produces no discernible male gametophyte defects, despite causing female-specific polar nuclei abnormalities and gibberellin-related dwarfism [34]. This disparity suggests either functional redundancy among APC/C subunits in the rice male germline, or lineage-specific rewiring of cell-cycle control that renders rice pollen less dependent on APC/C activity. Thus, even deeply conserved complexes like the APC/C can exhibit lineage-specific functional flexibility, underscoring the need for comparative analyses across angiosperms to distinguish universal requirements from derived dependencies.

Despite the APC/C’s indispensable role, a growing body of evidence argues that the core oscillator is necessary but insufficient to account for the full spatiotemporal precision of male gametogenesis. Two distinct functional sub-classes of E3 ligases have emerged that operate in concert with, or downstream of, the APC/C to ensure developmental fidelity.

First, a group of E3 ligases functions to clear meiosis-specific proteins that would otherwise interfere with post-meiotic development. The rice E3 ligase XBOS36 exemplifies this “meiotic cleaner” role. XBOS36 targets the Argonaute protein MEL1—which is essential for early meiotic progression but must be eliminated after meiosis—for ubiquitin-mediated degradation [26]. In the absence of *XBOS36*, persistent MEL1 leads to aberrant chromosome segregation and the formation of malformed microspores [26]. This principle—that dedicated E3s are required to purge the cell of meiosis-specific machinery before mitotic divisions can commence—is likely conserved, yet direct orthologs and analogous mechanisms in dicots remain poorly defined.

Second, a critical “brake-release” function is executed by E3 ligases that degrade cell-cycle inhibitors accumulated during meiosis. The RING-type E3 ligases RHF1a and RHF2a in *Arabidopsis* provide the canonical example. These E3s specifically target the cyclin-dependent kinase inhibitor ICK4/KRP6 for degradation during the meiotic-to-mitotic transition [23]. In the *rhf1a rhf2a* double mutant, ICK4/KRP6 accumulates, and microspores arrest prior to PMI—a phenotype that cannot be rescued by APC/C activity alone. A parallel pathway involving the shuttling factor RAD23B and the inhibitor KRP1 reinforces the notion that multiple, functionally overlapping UPS routes converge on the removal of division barriers [81], underscoring the evolutionary importance of this regulatory logic. The contrasting yet complementary roles of *XBOS36* (clearing meiotic machinery) and *RHF1a/2a* (removing mitotic brakes) illustrate how substrate-specific E3 ligases add essential layers of control atop the core APC/C oscillator.

Yet, this model immediately exposes a significant knowledge gap: the substrates for several genetically validated E3 ligases operating at later stages remain unknown. The APD1–4 family of transmembrane RING E3s is a case in point. Loss of APD function leads to a clear arrest at PMII, and interaction screens have identified associations with V-ATPase subunits and Rab GTPases, implicating the endomembrane system [24]. However, in the absence of identified ubiquitination targets, the precise molecular function of APD1–4 remains speculative. This “orphan E3” problem—where phenotype is established but substrate is elusive—represents a major bottleneck in the field, limiting our ability to move from descriptive genetics to predictive mechanistic models.

While the E3 ligases discussed above operate primarily within the developing microspore, additional functional tier of the UPS orchestrates the behavior of the surrounding sporophytic tissues, enforces spatial control over potentially deleterious proteins, and integrates external signals. This tier encompasses at least three mechanistically distinct modules.

The first module governs tapetal development and pollen wall formation. Anther dehiscence is a critical event in stamen development, enabling the release of mature pollen for successful pollination. This process is tightly regulated through coordinated programs of PCD [5,82]. The U-box E3 AtPUB4 is specifically expressed in the *Arabidopsis* tapetum, where it is required for timely programmed cell death and proper exine deposition [28]. Its wheat ortholog *TaPUB4*, however, has been functionally repurposed to influence pollen starch metabolism, with only partial conservation of function [29]. This suggests that the downstream target networks of U-box-type E3 ligases may have undergone significant lineage-specific remodeling following the divergence of monocots and dicots. This remodeling likely reflects adaptation to the distinct requirements for pollen development and energy metabolism in each lineage.

The second module enforces spatial control over mitochondrial proteins to prevent cytoplasmic male sterility (CMS). In CMS-WA rice, the mitochondrial-encoded protein WA352 is inherently toxic if allowed to accumulate. Its accumulation is prevented in most tissues by three F-box proteins—WIF1, WIF2, and WIF3—which recognize the N-terminus of WA352 and target it for ubiquitin-mediated degradation. Crucially, WIF expression is specifically downregulated in anthers during the microspore mother cell stage, creating a permissive window for WA352 accumulation that triggers tapetal PCD and pollen abortion [25]. This system exemplifies a recurring UPS theme: regulated degradation is used not merely for developmental timing, but as a precise spatial gatekeeper that restricts protein activity to specific cell types or developmental windows. Whether similar “degradation gating” mechanisms operate in non-CMS contexts remains an open question.

The third module integrates hormonal and environmental signals. The RING E3 ligase DAF in *Arabidopsis* promotes jasmonic acid (JA) biosynthesis—and thereby anther dehiscence—by activating transcription of DAD1, likely via degradation of an unknown transcriptional repressor [30]. The identity of this repressor remains a notable gap. Meanwhile, the rice E3 ligase CSIT1, a component of the ribosome-associated quality control (RQC) pathway, directly links temperature stress to male fertility [27]. In thermosensitive genic male sterile lines, elevated temperatures trigger CSIT1-mediated hyper-ubiquitination and degradation of catalases, impairing ROS scavenging in the tapetum and causing pollen abortion. This discovery not only provides a molecular framework for environment-sensitive fertility but also demonstrates that UPS components can function as “environmental rheostats,” tuning developmental outcomes in response to external conditions. Whether similar environmentally responsive UPS modules operate broadly across angiosperms, or whether *CSIT1* represents a specialized adaptation in rice, is a question of both fundamental and agricultural significance.

Finally, while the preceding examples focus on E3 ligases that catalyze ubiquitin conjugation, the reverse reaction—ubiquitin removal by deubiquitinating enzymes (DUBs)—is equally integral to UPS function. The ubiquitin-specific proteases UBP3 and UBP4 in *Arabidopsis* constitute a functionally redundant DUB subfamily that is essential for pollen development [83]. Single *ubp3* or *ubp4* mutants are phenotypically normal, but ubp3 ubp4 double-mutant pollen fails to complete PMII, producing grains with a single generative nucleus rather than two sperm cells. Rescue requires enzymatically active UBP3 or UBP4, confirming that deubiquitination activity per se is essential [83]. The substrates of UBP3/UBP4 remain unknown, leaving open the question of how ubiquitin removal interfaces with the E3-mediated regulatory networks—that converge on PMII progression.

In summary, the UPS governs male gametophyte development through a hierarchical architecture: a conserved APC/C oscillator drives the core cell-cycle engine; substrate-specific RING E3s execute distinct clearance functions—either removing meiosis-specific machinery (XBOS36) or clearing mitotic inhibitors (RHF1a/2a)—to permit developmental transitions; and tissue- or environment-sensing modules (AtPUB4/TaPUB4, DAF, WIF1–3, CSIT1) couple gametophyte development to its physiological context, enforce spatial protein restriction, and integrate external cues. Yet, this synthesis also illuminates persistent challenges. The substrates of APD1–4 and DAF remain unidentified, limiting mechanistic resolution. The regulatory logic underlying the functional divergence of orthologous E3s (e.g., AtPUB4 vs. TaPUB4) is poorly understood. And the extent to which environmentally responsive UPS pathways and spatial gating mechanisms are conserved across species awaits systematic investigation.

## 4. Role of the UPS in Female Gametophyte Development

**Figure 3:**
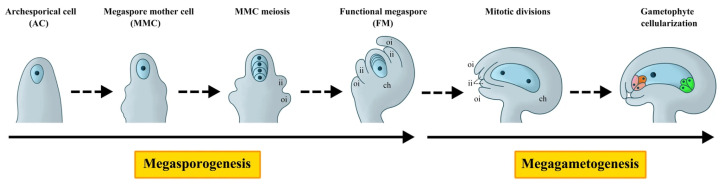
Different stages of female gametophyte development in Arabidopsis thaliana. Development of the female gametophyte comprises megasporogenesis and megagametogenesis. Prior to megasporogenesis, a subepidermal cell at the apex of the ovule primordium differentiates into an archesporial cell (AC), which develops directly into a megaspore mother cell (MMC). Megasporogenesis is initiated by meiotic division of the MMC, producing four haploid megaspores. Only the chalazal-most megaspore survives as the functional megaspore (FM), while the three micropylar megaspores degenerate. During megagametogenesis, the FM undergoes three rounds of mitosis accompanied by nuclear migration and cellularization, resulting in a mature female gametophyte (FG) or embryo sac. ii, inner integument; oi, outer integument; ch, chalaza.

The second stage of female gametophyte development commences from the functional megaspore and proceeds through a series of mitotic divisions, generating two-nucleate, four-nucleate, and eight-nucleate embryo sacs, ultimately culminating in a mature seven-celled, eight-nucleate structure [84,85,86] (Figure 3). During this process, the antipodal cells gradually degenerate, while the central cell and the egg cell participate in double fertilization, giving rise to the endosperm and the embryo, respectively [87]. The three sequential nuclear mitotic divisions are critical, and their precise execution depends on the timely degradation of key cell cycle proteins, particularly during the metaphase-to-anaphase transition and the progression of anaphase itself. The most robustly established function of the UPS in female gametophyte development centers on the APC/C. Pioneering work in *Arabidopsis* identified *NOMEGA*, encoding the APC6/CDC16 subunit, as essential for female gametophyte progression beyond the two-nucleate stage (FG2), with mutant embryo sacs exhibiting complete mitotic arrest and failure of central vacuole formation [31]. This phenotype was mechanistically linked to the accumulation of Cyclin B, directly confirming compromised APC/C ubiquitin ligase activity [31]. Subsequent analyses of additional subunits—APC1 and APC4—reinforced this model, revealing that loss of either component leads to strikingly similar defects: nuclear degradation during megagametogenesis, loss of embryo sac polarity, and pleiotropic embryonic arrest [32,33]. The synergistic enhancement of fertility defects in *apc1 apc4* double mutants provides genetic evidence that these subunits act in concert to maintain the structural and functional integrity of the APC/C holocomplex. The observation that mutations in different APC/C subunits produce overlapping yet non-identical phenotypes likely reflects their distinct structural roles: APC1 functions as a core scaffold essential for complex integrity, APC4 serves as a linker between submodules, and APC6/CDC16 participates in substrate recognition—such that subunit-specific lesions differentially perturb complex assembly, activity, or target specificity. The synergistic enhancement of fertility defects in *apc1 apc4* double mutants provides genetic evidence that these subunits act in concert to maintain the structural and functional integrity of the APC/C holocomplex.

This body of work has led to a strong consensus: the APC/C functions as an indispensable mitotic driver in the female germline, just as it does in pollen. The core logic—APC/C-mediated degradation of mitotic cyclins to permit metaphase-to-anaphase transitions—appears conserved across both gametophytic lineages. However, a critical comparison with the male gametophyte reveals important sex-specific nuances. In *Arabidopsis*, APC/C dysfunction produces broadly similar cell-cycle arrest phenotypes in both sexes. Yet, the female gametophyte exhibits additional vulnerabilities not observed in pollen, including disrupted polarity establishment and aberrant auxin gradient formation [32,33]. This suggests that the APC/C in the embryo sac is integrated into a more complex regulatory network involving hormone signaling and spatial patterning cues [22]. Furthermore, the rice *apc6* mutant illustrates that subunit requirements can differ dramatically between tissues and species: male gametophyte development proceeds normally despite female-specific polar nuclei defects and sporophytic gibberellin insensitivity [34], implying either functional redundancy among APC/C subunits in the male germline or lineage-specific rewiring of downstream targets.

While the APC/C dominates the female UPS literature, several studies have begun to illuminate a broader regulatory repertoire. Importantly, some UPS modules first characterized in the male germline also function in the female gametophyte, reinforcing the concept of shared regulatory toolkits redeployed in distinct developmental contexts. The RING-type E3 ligases RHF1a and RHF2a, discussed in Section 3 for their essential role in degrading ICK4/KRP6 during male PMI, are equally critical for female gametophyte development. In the *rhf1a rhf2a* double mutant, embryo sac development arrests predominantly at FG1—the one-nucleate stage immediately following functional megaspore specification—demonstrating that RHF1a/2a-mediated degradation of cell-cycle inhibitors is a conserved prerequisite for mitotic entry in both female and male gametogenesis [23]. This shared requirement underscores a fundamental principle: plants deploy the same UPS-mediated “brake-release” machinery to clear meiosis-accumulated inhibitors.

In addition to these shared modules, a small number of UPS components have been identified with female-biased or female-specific functions, providing glimpses into regulatory innovations unique to the embryo sac. The ubiquitin-conjugating enzyme UBC22 offers a particularly instructive example. Unlike canonical E2 enzymes that catalyze Lys48-linked ubiquitin chains targeting substrates for proteasomal degradation, UBC22 mediates atypical Lys11-linked ubiquitination [59]. Loss of *UBC22* function results specifically in degeneration of the functional megaspore and female gametes, with no reported effect on male gametophyte viability [59]. This example reinforces a fundamental concept introduced in Section 2: the distinct topologies of polyubiquitin chains—K48, K63, and K11—encode different functional messages. The female-specific requirement for *UBC22* illustrates how a particular chain topology (K11-linked) is essential in the female germline. Whether K11-linked chains are similarly required in the male germline, perhaps catalyzed by as-yet-unidentified E2 or E3 enzymes, remains an open question. The specific substrates modified by UBC22-catalyzed K11 chains and the molecular basis for their female germline-specific importance also await further investigation.

From the above, it is evident that male and female gametophyte development relies heavily on APC/C-mediated degradation of cyclins (e.g., *NOMEGA*, *APC1*, *APC4*). However, female gametogenesis is characterized by a stricter requirement for synchrony of nuclear divisions, a heightened sensitivity to polarity cues that guide embryo sac formation, and a relatively sparse regulatory environment of female-specific E3 ligases. Far fewer female-specific E3 ligases have been identified to date compared to their male counterparts, suggesting the existence of an as-yet-uncovered regulatory network.

## 5. The UPS in Male–Female Gametophyte Interactions

### 5.1. Regulation of Pollen Tube Growth by the UPS

During double fertilization in angiosperms, the pollen tube plays a pivotal role [88]. As an extension of the male gametophyte, it serves as the sole conduit for delivering two immotile sperm cells from the pollen grain, through the pistil, and into the embryo sac, thereby enabling fertilization of both the egg cell and the central cell. This directional growth relies on intricate signaling mechanisms and cell–cell communication, including guidance by chemical attractants secreted by the pistil and the regulation of polar growth at the pollen tube tip [89,90,91] Upon arrival, the pollen tube enters a synergid, where it ruptures to release the two sperm cells. This discharge sets the stage for double fertilization: one sperm fuses with the egg cell to form the zygote, while the other fuses with the central cell to initiate endosperm development [92]. This highly coordinated process is fundamental to successful seed formation and thus constitutes a core event in angiosperm sexual reproduction.

Evidence accumulated over the past two decades indicates that the UPS participates in regulating multiple facets of this process, although the depth of mechanistic understanding varies considerably across different experimental systems. The field’s trajectory has progressed through distinct phases: from initial pharmacological observations implicating proteasome activity, to the genetic identification of specific E3 ligases required for normal pollen tube growth, and more recently, to the recognition that ubiquitin may function independently of proteolysis in pollen–pistil interactions. However, a recurring limitation is the substantial gap between phenotypic observation and mechanistic resolution—a gap that mirrors the “orphan E3” problem highlighted in earlier sections.

The earliest evidence linking the UPS to pollen tube growth came from pharmacological studies using proteasome inhibitors such as MG132. In kiwifruit (*Actinidia deliciosa*), MG132 treatment blocked pollen germination and arrested the growth of established tubes, causing tip swelling, branching, and the accumulation of ubiquitinated proteins [93]. These observations established that proteasome activity is continuously required to maintain the apical “clear zone” and the polarized cytoplasmic organization of the growing pollen tube. Subsequent work in spruce (*Picea*) pollen tubes extended these findings, revealing that proteasomal dysfunction induces ER-derived vacuolization, disrupts both actin and microtubule cytoskeletons, and reduces apical cell wall components [94]. Collectively, these pharmacological studies converged on a consistent finding: proteasome inhibition perturbs pollen tube growth and polarity. However, because proteasome inhibitors globally block degradation, these experiments could not identify the specific E3 ligases or substrates responsible, nor could they distinguish between direct regulatory roles and indirect effects stemming from general proteotoxic stress. This limitation motivated subsequent genetic approaches.

The transition from pharmacology to genetics has identified specific E3 ligases whose loss or overexpression affects pollen tube growth, yet mechanistic understanding remains fragmentary. Two RING-type E3 ligases—rice PTB1 and lily LIANK—illustrate both the progress made and the challenges that persist. In rice, the *PTB1* (POLLEN TUBE BLOCKED 1) gene encodes a RING-type E3 ligase that functions as a maternal factor in the style to promote pollen tube growth; natural variation in *PTB1* is associated with altered seed-setting rates [35]. Notably, *PTB1* expression is suppressed by temperature stress, suggesting a potential link between environmental signals and UPS-dependent regulation. However, the direct ubiquitination substrates of PTB1 remain unidentified, limiting understanding of how it facilitates pollen tube elongation at the molecular level. Similarly, the lily RING-type E3 ligase LIANK localizes to membrane organelles and is required for normal pollen germination and tube growth; its overexpression causes ectopic budding and tip swelling, whereas knockdown impairs germination [36]. Despite the clear phenotypic evidence for LIANK’s involvement in membrane dynamics, neither its substrates nor the specific membrane compartments it regulates have been defined. These two cases exemplify a broader pattern: genetic studies have validated the importance of specific E3 ligases for normal pollen tube growth, but for most—including PTB1 and LIANK—the biochemical pathway from E3 activity to growth regulation remains uncharacterized. The identification of their substrates is a prerequisite for determining whether these E3 ligases act as dedicated regulators of tip growth or function more broadly in cellular homeostasis.

A particularly intriguing development has been the recognition that ubiquitin can function in pollen–pistil interactions independently of its classical role as a proteolytic tag. In lily, exogenous free ubiquitin significantly enhances pollen tube adhesion to an in vitro stylar matrix, acting synergistically with the pistil-secreted cysteine-rich adhesin SCA [95]. In this context, ubiquitin does not signal degradation; rather, it appears to promote the endocytic trafficking of SCA into multivesicular bodies, thereby modulating the adhesive interface between the pollen tube and the stylar tract [95]. This discovery indicates the non-proteolytic function of ubiquitin in adhesion—and potentially in other aspects of pollen–pistil communication—warrants systematic investigation. This non-proteolytic function of ubiquitin likely involves K63-linked polyubiquitin chains, which serve as sorting signals for endocytic trafficking independently of the 26S proteasome [57,58]. The observation that exogenous ubiquitin promotes pollen tube adhesion thus exemplifies how distinct ubiquitin chain topologies can encode fundamentally different signaling outcomes in reproductive contexts. The observation that exogenous ubiquitin promotes pollen tube adhesion thus exemplifies how distinct ubiquitin chain topologies can encode fundamentally different signaling outcomes in reproductive contexts. And if so, what are receptors and downstream effectors of ubiquitin in the pollen tube or stylar apoplast?

### 5.2. UPS-Mediated Regulation of Self-Incompatibility

Self-incompatibility (SI) systems, found in approximately 50% of flowering plant species, prevent self-fertilization. Among these, the S-RNase-based mechanism is particularly well-characterized [96]. In this system, ubiquitination serves as a central molecular switch that precisely regulates the fertilization outcome [97,98]. This mechanism is governed by the polymorphic S-locus, which encodes stylar-expressed S-RNases that function as cytotoxins. These RNases inhibit the growth of pollen tubes carrying the same S-haplotype, thereby preventing self-fertilization in many species of Plantaginaceae, Solanaceae, and Rosaceae [99,100,101]. Upon cross-pollination, pollen-expressed SLF (S-Locus F-box) proteins act as specificity determinants and assemble into SCF ubiquitin ligase complexes. These complexes selectively recognize and ubiquitinate non-self S-RNases, targeting them for proteasomal degradation. This degradation alleviates the cytotoxic inhibition and facilitates compatible pollen tube growth and successful fertilization [102,103,104]. In contrast, during self-pollination, self S-RNases evade recognition and degradation. Instead, they undergo phase separation to form cytoplasmic condensates, which disrupt the actin cytoskeleton and redox homeostasis. These events ultimately trigger programmed cell death in self-pollen tubes, enforcing the self-incompatibility response [100,105,106].

In the self-incompatibility (SI) system of Lilium, the SCF complex—a central component of the ubiquitination machinery—plays a critical role in determining pollen tube fate. Three pollen-specific SKP1-like genes, *LSK1*, *LSK2* and *LSK3*, are highly expressed during late pollen development and pollen tube elongation. The encoded LSK proteins contain a conserved N-terminal Cullin1-binding domain and a C-terminal F-box protein-binding domain, confirming their structural role as canonical SKP1 adaptors within SCF complexes. Functional assays demonstrate that these LSK proteins interact with the Lilium Cullin1 homolog and functionally complement a yeast skp1 mutant, validating them as bona fide SCF components. To investigate their function, a dominant-negative interference strategy was employed using truncated LSK variants (LSKΔ-GFP) that lack the N-terminal Cullin1-binding domain. These mutants can still bind F-box proteins via their intact C-terminus but fail to assemble into functional SCF complexes. Overexpression of LSK2Δ and LSK3Δ significantly inhibited pollen tube elongation both in vivo and in vitro, and this inhibitory effect was markedly more pronounced under self-pollination conditions. These findings provide direct evidence that LSK-containing SCF complexes positively regulate pollen tube growth, likely by mediating the degradation of specific substrates, and suggest that these complexes are deeply involved in the regulation of self-incompatibility in Lilium [37]. In striking contrast to this multi-subunit assembly, research on the SI system of Brassicaceae has revealed a more streamlined “molecular switch” governed by the single-subunit U-box E3 ligase, ARC1. In this well-characterized module, interaction with self-pollen triggers an S-haplotype-specific ligand to activate the stigmatic receptor kinase SRK [107]. Activated ARC1 then specifically targets Exo70A1 for ubiquitination and proteasomal degradation. Exo70A1, a core subunit of the exocyst complex, serves as a positive regulator of pollen acceptance by directing vesicle trafficking to the plasma membrane—a process essential for pollen hydration and compatible pollination [108]. Its degradation by ARC1 effectively dismantles this secretory machinery, blocking self-pollen acceptance. The biological necessity of this pathway is reinforced by both functional and evolutionary evidence: knockdown of *ARC1* in the self-incompatible species *Arabidopsis lyrata* attenuates the SI response, and in self-compatible Brassicaceae species (e.g., *Arabidopsis thaliana*), ARC1 is consistently lost or pseudogenized, indicating that the inactivation of ARC1 represents a key evolutionary step in the transition from obligate outcrossing to self-compatibility [39].

These two distinct self-incompatibility (SI) systems converge on a central principle by which the ubiquitin–proteasome system (UPS) mediates intraspecific reproductive isolation: the recognition of “non-self” versus the elimination of “self.” In SI, the SCF complex acts as a sensor of non-self, selectively degrading cytotoxic S-RNases of incompatible origin and thereby promoting fertilization. In the Brassicaceae-type SI system, by contrast, a U-box E3 ligase (ARC1) is activated upon self-recognition and targets compatibility factors for degradation, effectively blocking fertilization. Although the underlying regulatory logic appears reversed—one system positively regulating outcrossing while the other negatively regulating self-fertilization—both ultimately exploit the precision of UPS-mediated proteolysis as a molecular switch that determines the fate of the pollen tube. Moreover, the recent identification of an interaction between PbrARI2.3 and brassinosteroid signaling in pear suggests that the UPS does not act in isolation. The protein stability of PbrBZR1—a key transcription factor in the brassinosteroid (BR) signaling pathway—is dynamically regulated by the pollen-specific E3 ligase PbrARI2.3. Under SI conditions, accumulated PbrARI2.3 specifically recognizes the dephosphorylated (active) form of PbrBZR1, targeting it for ubiquitination and subsequent 26S proteasomal degradation. The resulting depletion of PbrBZR1 suppresses the expression of *PbrEXLA3*, which encodes an expansin-like protein required for pollen tube elongation. This downregulation of *PbrEXLA3* compromises cell wall extensibility, ultimately inhibiting pollen tube growth [38]. This example illustrates how UPS-mediated degradation can integrate with broader signaling networks to enforce the self-incompatibility response.

In addition to the well-established ARC1-mediated pathway, recent studies have suggested that other classes of E3 ligases, particularly U-box-type family members, may contribute to the intricate regulatory networks governing SI in Brassicaceae. For instance, a gene encoding a U-box-type E3 ubiquitin ligase, designated *BoPUB7*, was identified in *Brassica oleracea* and found to be specifically induced by self-pollination. Its transcript levels increase approximately threefold in stigmas as early as 30 min post-pollination. Functional analyses in *Arabidopsis* revealed that ectopic overexpression of *BoPUB7* significantly inhibits pollen germination, whereas T-DNA insertion mutants of the putative *Arabidopsis* homolog display normal germination. These observations suggest that PUB7 possesses the capacity to act as a negative regulator of pollen germination, at least in a heterologous context. Furthermore, protein–protein interaction screening demonstrated that PUB7 interacts with UBA2b, a protein containing an RNA recognition motif (RRM). This interaction raises the possibility that PUB7 may mediate SI signaling through ubiquitination of this putative downstream component. Collectively, these findings introduce the PUB7-UBA2b module as a potential additional regulatory branch of the SI signaling network, operating alongside the well-established ARC1-Exo70A1 pathway. This discovery further underscores the complexity of the regulatory framework, in which distinct classes of E3 ligases collaboratively fine-tune the self-incompatibility response [40].

It is noteworthy that certain reproductive processes, such as the Ca^2+^-mediated programmed cell death pathway in Papaveraceae self-incompatibility [96], appear to operate largely independently of canonical UPS-mediated degradation, highlighting the diversity of regulatory strategies employed by flowering plants and underscoring that the UPS, while pervasive, is but one component of a larger regulatory toolkit.

### 5.3. The UPS in Reproductive Isolation and Evolutionary Adaptation

In addition to its well-characterized role in fine-tuning intraspecific reproductive development, the ubiquitin–proteasome system has emerged as a key player in mediating reproductive isolation at the interspecific and subspecific levels, thereby influencing plant evolutionary trajectories and speciation. A recent study on temperature-sensitive reproductive isolation between the two Asian cultivated rice subspecies *indica* (*Oryza sativa* ssp. *indica*) and *japonica* (*Oryza sativa* ssp. *japonica*) has uncovered a three-gene module (*SaF*, *SaM*, and *SaFL*) that functions as a “pollen killer–protector” system. This module regulates pollen fertility through UPS-mediated protein degradation and establishes a reproductive barrier associated with latitudinal adaptation.

In heterozygous backgrounds, proteins encoded by the *indica* alleles *SaF^+^* and *SaM^+^* assemble into a mitochondrial complex that functions as part of an SCF-type E3 ubiquitin ligase. This complex specifically recognizes and ubiquitinates the mitochondrial reactive oxygen species (ROS) scavenger COX11, targeting it for degradation via the 26S proteasome [41]. The resulting loss of COX11 leads to excessive ROS accumulation, which in turn causes pollen abortion in individuals carrying the *japonica* allele *Sa-j*, thereby executing the “killer” function. Notably, a protective mechanism operates within this system: the *indica*-derived *SaFL^+^* acts as a potent protector that competitively binds SaM^+^, disrupting killer complex assembly and restoring pollen fertility. In contrast, the *japonica* allele *SaFL*^−^ encodes a weak protector whose function is temperature-dependent—it effectively binds SaM^+^ and attenuates killer complex activity only under high-temperature conditions, partially restoring pollen fertility [41].

From an evolutionary perspective, the *Sa* system exemplifies a classic Bateson–Dobzhansky–Muller (BDM) incompatibility, in which postzygotic reproductive isolation arises from deleterious epistatic interactions between alleles that have diverged in geographically separated populations [109,110]. Unlike prezygotic barriers that prevent mating, BDM incompatibilities reduce hybrid fitness after fertilization and are considered a fundamental engine of plant speciation. Evolutionary analyses suggest that the *Sa* locus originated in ancient wild rice and underwent functional diversification during the range expansion of cultivated rice from tropical to temperate environments. Haplotypes carrying either a non-functional killer complex or a strong protector were selectively fixed in *japonica* and *indica* populations, respectively [41]. This latitudinally associated divergence suggests that the *Sa* system not only enforces reproductive isolation but may have been shaped by local adaptation to temperature regimes—a scenario consistent with the hypothesis that ecological selection can drive the evolution of hybrid incompatibilities during allopatric or parapatric divergence. Notably, to date, functionally analogous UPS-mediated reproductive isolation systems have not been reported in other plant taxa, raising the intriguing question of whether the *Sa* module represents a unique evolutionary innovation in rice or whether similar mechanisms await discovery in other species. Collectively, these findings reveal a previously unrecognized role for the UPS in regulating subspecific reproductive interactions and provide a compelling molecular paradigm for understanding how UPS-mediated protein degradation can be co-opted to drive reproductive isolation and speciation in plants.

## 6. The UPS in Embryogenesis and Endosperm Development

Successful double fertilization initiates two genetically distinct developmental programs: the embryo, which establishes the next generation, and the endosperm, a terminally differentiated nutritive tissue that supports embryogenesis. Both processes depend critically on precisely timed protein degradation events mediated by the UPS. However, the regulatory logic deployed in these post-fertilization stages differs fundamentally from that operating during gametophyte development. Whereas gametogenesis relies on the UPS primarily to drive cell-cycle progression and clear division barriers, embryogenesis and endosperm development recruit the UPS for functionally distinct purposes: decoding spatial auxin gradients to pattern the embryonic axis, and enforcing the epigenetic silencing of imprinted genes that governs endosperm development.

The formation of the apical–basal axis during embryogenesis depends on spatially restricted auxin maxima, which are generated by polar auxin transport and interpreted through UPS-dependent proteolysis. The core UPS component in this pathway is the F-box protein TIR1, which functions as the substrate recognition subunit of the SCF E3 ligase complex [42,43]. In cells where auxin concentration exceeds a critical threshold, the SCF^TIR1 complex binds to BODENLOS (BDL)/IAA12—an Aux/IAA family transcriptional repressor—and targets it for proteasomal degradation [111,112]. The resulting loss of BDL/IAA12 relieves inhibition of MONOPTEROS (MP)/ARF5, a transcription factor required for root pole specification [111,112]. The functional integrity of this pathway depends not only on the substrate recognition subunit TIR1 but also on the invariant structural core of the SCF complex. The *axr6* mutant, which encodes a defective CULLIN1 (CUL1) scaffold subunit, disrupts SCF complex assembly and consequently impairs SCF^TIR1-mediated degradation of Aux/IAA proteins [44]. The *axr6* phenotype is markedly more severe than that of *tir1* because CUL1 is shared by all SCF-type E3 ligases. This contrast illustrates a recurring principle: substrate specificity is diversified through interchangeable adaptors, whereas the core catalytic machinery remains centralized and indispensable—a design that confers both functional versatility and system-wide robustness. Meanwhile, as introduced in Section 4, the distorted auxin distribution observed in *apc4* embryos suggests that the APC/C influences embryogenesis by modulating auxin transport or signaling, although the precise molecular connections remain to be elucidated [32,33]. This convergence of multiple E3 complexes on auxin-mediated patterning illustrates a theme: the UPS governs developmental processes not through a single linear pathway, but through a network of functionally distinct complexes acting from different regulatory angles.

The UPS interfaces with Polycomb Repressive Complex 2 (PRC2) at multiple regulatory nodes, employing both ubiquitin conjugation and deubiquitination to fine-tune this central epigenetic machinery. On the conjugation side, the F-box protein UCL1 functions as the substrate recognition subunit of an SCF E3 ligase that directly targets CURLY LEAF (CLF)—a SET-domain catalytic subunit of certain PRC2 complexes—for ubiquitin-mediated degradation [48]. In the endosperm, this activity is particularly critical: the essential PRC2 catalytic subunit MEDEA (MEA) is a close homolog of CLF, and because both can interact with the same PRC2 scaffold, CLF accumulation competes with MEA and disrupts functional FIS-PRC2 complex formation. UCL1, prominently expressed in the endosperm shortly after fertilization, selectively clears CLF to ensure MEA-containing complexes predominate [48]. On the deubiquitination side, the ubiquitin-specific protease UBP26 removes ubiquitin from histone H2B, thereby facilitating PRC2-mediated deposition of H3K27me3 at imprinted loci such as *PHERES1* [113]. In the absence of UBP26, persistent H2B ubiquitination impedes PRC2-mediated silencing [113]. Beyond SCF complexes, CRL4-DCAF (DDB1-CUL4 ASSOCIATED FACTORS) modules add a further layer of control [45,114]. One such DCAF, WDR55, has been demonstrated to interact with DDB1A and is essential for both reproductive and vegetative development in *Arabidopsis* [45]. WDR55 is essential for embryonic apical patterning, and its mutant phenotypes resemble those of auxin transport mutants [45,46]. This phenotypic spectrum positions WDR55 and the CRL4 complex as important regulators of auxin-mediated developmental processes, potentially acting in parallel with or downstream of SCF-mediated Aux/IAA degradation. Another DCAF, MULTICOPY SUPPRESSOR OF IRA1 1 (MSI1) serves dual roles as both a CRL4 substrate receptor and a core PRC2 subunit [49], physically associates with DDB1A and is required to maintain parental imprinting of the MEDEA locus in the endosperm [115]. Together, these activities illustrate a broader principle: the UPS governs PRC2-dependent epigenetic regulation through the coordinated action of E3 ligases and DUBs, which together determine both the composition of PRC2 complexes and their efficacy at target loci. Beyond substrate-specific regulation, the UPS must also maintain its own functional integrity during the intense protein turnover that accompanies early embryogenesis. The reversible nature of ubiquitination—governed by the opposing activities of E3 ligases and deubiquitinating enzymes (DUBs)—is equally critical for post-fertilization development. The ubiquitin-specific protease UBP14 provides a compelling case study. Loss of *AtUBP14* function results in embryonic arrest at the globular stage, accompanied by the accumulation of multi-ubiquitin chains, indicating that UBP14-mediated ubiquitin recycling is essential for early embryo development [116]. The *ubp14* phenotype closely resembles that of proteasome subunit mutants, positioning UBP14 as a key component of the cellular ubiquitin supply chain during embryogenesis. Importantly, the E3 ligases MAC3A and MAC3B have recently been shown to ubiquitinate UBP14 itself, targeting it for degradation and thereby establishing a regulatory feedback loop that controls UBP14 abundance [47]. This discovery illustrates a broader principle: the UPS operates as a network of interconnected regulatory loops in which E3 ligases and DUBs mutually regulate each other’s activity.

## 7. Conclusions and Future Perspectives

The diverse UPS functions surveyed in this review can be organized into a hierarchical framework comprising three regulatory layers, which collectively establish the UPS as a central regulator of cell-cycle progression, tissue-specific PCD, hormone signaling, and epigenetic control in plant reproduction. At the core, the APC/C functions as a deeply conserved cell-cycle oscillator that drives the meiotic and mitotic divisions in both male and female gametophytes (Section 3 and Section 4). Operating independently of this oscillator is a layer of substrate-specific E3 ligases and deubiquitinating enzymes that execute context-dependent clearance or stabilization events—removing meiotic machinery (XBOS36), degrading cell-cycle inhibitors (RHF1a/2a, RAD23B), enabling sperm cell formation (APD1–4, UBP3/4), decoding positional information during embryogenesis (SCF^TIR1), remodeling PRC2 complexes (UCL1, UBP26), and maintaining ubiquitin homeostasis (UBP14). A third layer comprises tissue- and environment-responsive modules—including tapetum-specific E3s (AtPUB4) for PCD, hormone-integrated ligases (DAF, PbrARI2.3) for signaling, spatially gated CMS regulators (WIF1–3), and temperature-sensing quality control machinery (CSIT1)—that couple internal developmental programs to external signals. Disruption of these UPS components consistently leads to sterility, seed abortion, or reproductive failure.

Several trends emerging from the comparative organization of UPS components in Table 1 reinforce this framework. APC/C subunits are exclusively associated with gametophyte cell-cycle control in both sexes, consistent with their foundational role. RING-type E3s are predominantly involved in substrate-specific clearance during male mitotic transitions, whereas U-box and SCF-type E3s are enriched in tissue-specific and environment-responsive modules across sporophytic tissues and post-fertilization stages. CRL4-DCAF complexes (WDR55, MSI1) appear restricted to embryogenesis and endosperm, suggesting a specialized role in post-fertilization chromatin regulation. A pronounced asymmetry in mechanistic resolution is also evident: many E3 ligases—including APD1–4, DAF, AtPUB4/TaPUB4, and WDR55—lack biochemically defined substrates, whereas those with validated targets are concentrated in few intensively studied pathways. Cross-species comparisons further highlight lineage-specific divergence, exemplified by the contrasting requirements of OsAPC6 in rice versus *Arabidopsis* APC/C subunits, and the functional repurposing of AtPUB4 and TaPUB4 between tapetal PCD and pollen starch metabolism. Overall, Table 1 reveals a stage-specific regulatory shift—from cell-cycle drive in gametophytes to signal integration and epigenetic control in post-fertilization stages.

However, realizing the full potential of this framework—particularly mapping the complete inventory of substrate-specific E3s and their targets—requires overcoming several persistent knowledge gaps. A critical bottleneck recurring throughout this review is the substantial gap between genetic validation and mechanistic understanding. Many E3 ligases discussed above—including APD1–4, DAF, AtPUB4/TaPUB4 and WDR55—exhibit clear mutant phenotypes but lack biochemically defined substrates. For these “orphan E3s”, the integration of AI-powered structural prediction (e.g., AlphaFold-Multimer) with proximity labeling and ubiquitinome profiling in reproductive tissues offers a direct path toward substrate identification. However, their utility for plant UPS research faces notable limitations: accuracy drops substantially for the small, transient interfaces characteristic of many E3–substrate contacts; post-translational modifications such as phosphorylation—often essential for substrate recognition—are not modeled; and plant-specific E3s frequently lack the deep evolutionary sequence alignments required for high-confidence predictions. AlphaFold should therefore be viewed not as a standalone solution, but as a hypothesis-generating tool whose predictions require rigorous validation through proximity-labeling proteomics and in vitro ubiquitination assays.

Beyond cataloging substrates, the examples of CSIT1 (temperature-responsive RQC) and the rice Sa system (UPS-mediated reproductive isolation) highlight the need to understand how the UPS integrates environmental and evolutionary signals into reproductive decisions. Multi-condition ubiquitinome profiling under defined stress regimes, combined with phylogenetic analyses of E3 families across monocots and dicots, will clarify both the environmental responsiveness and lineage-specific functional drift of conserved UPS modules. Finally, the translational potential of UPS manipulation—already demonstrated by CSIT1 in two-line hybrid breeding and the Sa system in intersubspecific barriers—warrants the development of targeted protein degradation tools (e.g., auxin-inducible degron systems) and precision genome editing of substrate-binding domains in crop E3 ligases. By directly linking each methodological advance to the specific unresolved questions identified in this review, future research can transform our understanding of the UPS from a descriptive catalog of reproductive phenotypes into a predictive and actionable mechanistic framework.

## Figures and Tables

**Table 1:** E3 Ligase Types and Substrates in Flowering Plant Reproductive Development.

E3 Type	Protein or Complex	Reproductive Process	Specific Stage/Function	Targets	Organism	References
CRLs	APC8/APC13	Male gametophyte	Meiotic chromosome segregation; PMI/PMII progression	CYCB1;1	*Arabidopsis thaliana*	[20,21,22]
RING	RHF1a/RHF2a	Male gametophyte	Post-meiotic mitotic entry	ICK4/KRP6	*Arabidopsis thaliana*	[23]
RING	APD1–4	Male gametophyte	PMII progression	-	*Arabidopsis thaliana*	[24]
SCF	WIF1/WIF2/WIF3	Male gametophyte	CMS suppression; spatiotemporal control of WA352	WA352	*Oryza sativa*	[25]
CRLs	XBOS36	Male gametophyte	Meiosis; MEL1 degradation	MEL1	*Oryza sativa*	[26]
RING	CSIT1	Male gametophyte	Ribosome-associated quality control; temperature response	CATs	*Oryza sativa*	[27]
U-box	AtPUB4	Male gametophyte	Tapetum PCD; exine formation	-	*Arabidopsis thaliana*	[28]
U-box	TaPUB4	Male gametophyte	Pollen starch accumulation	-	*Triticum aestivum* L.	[29]
RING	DAF	Male gametophyte	Anther dehiscence; JA biosynthesis	-	*Arabidopsis thaliana*	[30]
CRLs	NOMEGA	Female gametophyte	FG2 mitotic progression	-	*Arabidopsis thaliana*	[31]
CRLs	APC1/APC4	Female gametophyte	Female gametogenesis; embryogenesis	-	*Arabidopsis thaliana*	[32,33]
CRLs	OsAPC6	Female gametophyte	Polar nuclei formation; GA signaling integration	-	*Oryza sativa*	[34]
RING	PTB1	Pollen tube growth	Pollen tube elongation; temperature stress response	-	*Oryza sativa*	[35]
RING	LIANK	Pollen tube growth	Membrane dynamics; tip growth	-	*Lilium*	[36]
CRLs	LSK1/LSK2/LSK3	Pollen tube growth/SI	SCF complex assembly; pollen tube elongation	-	*Lilium*	[37]
RBR	PbrARI2.3	Self-incompatibility	BR signaling integration; SI-induced pollen tube inhibition	PbrBZR1	*Pyrus* spp.	[38]
U-box	ARC1	Self-incompatibility	SI response; stigmatic rejection	Exo70A1	*Arabidopsis lyrata*	[39]
U-box	BoPUB7	Self-incompatibility	Stigmatic SI signaling	UBA2b	*Brassica oleracea*	[40]
CRLs	SaF/SaM/SaFL	Reproductive isolation	ntersubspecific pollen killer	COX11	*Oryza sativa*	[41]
SCF	TIR1	Embryogenesis	Embryonic axis formation; auxin-dependent Aux/IAA degradation	BDL/IAA12	*Arabidopsis thaliana*	[42,43]
SCF	AXR6/CUL1	Embryogenesis	SCF complex scaffold; Aux/IAA degradation	-	*Arabidopsis thaliana*	[44]
CRL4 (DCAF)	WDR55	Embryogenesis	Apical patterning; bilateral symmetry establishment	-	*Arabidopsis thaliana*	[45,46]
RING	MAC3A/MAC3B	Embryogenesis	UBP14 ubiquitination; organ size control	UBP14	*Arabidopsis thaliana*	[47]
SCF	UCL1	Endosperm development	PRC2 complex remodeling; CLF degradation	CLF	*Arabidopsis thaliana*	[48]
CRL4 (DCAF)	MSI1	Endosperm development	PRC2 subunit; MEDEA imprinting maintenance	-	*Arabidopsis thaliana*	[49]

## Data Availability

The original contributions presented in this study are included in the article.
